# Lessons Learned From a COVID-19 Biohazard Spill During Swabbing at a Quarantine Facility

**DOI:** 10.1017/dmp.2020.436

**Published:** 2020-11-05

**Authors:** Oren Mayer, Tiffany Pfundt, Gamola Z. Fortenberry, Brian H. Harcourt, William A. Bower

**Affiliations:** 1 Centers for Disease Control and Prevention, Atlanta, Georgia, USA; 2 Laboratory Leadership Service assigned to NCEZID, Centers for Disease Control and Prevention, Atlanta, Georgia, USA; 3 Food and Drug Administration, Silver Spring, Maryland, USA; 4 US Department of Agriculture, Food Safety and Inspection Service, Washington DC

**Keywords:** SARS-CoV-2, COVID-19, cruise ships, quarantine facility, biohazard mitigation, and biocontainment

## Abstract

The need for increased testing for severe acute respiratory syndrome coronavirus 2 (SARS-CoV-2), the virus that causes coronavirus disease 2019 (COVID-19), has resulted in an increase of testing facilities outside of traditional clinical settings and sample handling by individuals without appropriate biohazard and biocontainment training. During the repatriation and quarantine of passengers from the *Grand Princess* cruise ship at a US military base, biocontainment of a potentially infectious sample from a passenger was compromised. This study describes the steps taken to contain the spill, decontaminate the area, and discusses the needs for adequate training in a biohazard response.

Adequate testing capacity is fundamental to controlling the coronavirus disease 2019 (COVID-19) pandemic.^1^ Demand for severe acute respiratory syndrome coronavirus 2 (SARS-CoV-2) sampling in nontraditional healthcare settings presents unique challenges for the safety of staff collecting specimens.^2^ To meet this demand, the scope of those performing sampling activities continues to expand to individuals without appropriate background in biohazard mitigation or biosafety training.^3-6^ Without proper training, this intersection of field response, infection prevention and control, and worker safety serves as a weak point to both the safety and quality of the response. An example of this occurred during sample collection from quarantined individuals from the *Grand Princess* cruise ship at a US military base. The resulting biohazard incident drove deeper discussion into proper sample handling, broader implications for improving the safety and practices of those working with COVID-19 samples in the field, and the importance of including those with biohazard and biocontainment training in these situations.

## Narrative

*Grand Princess* passengers arrived for quarantine at the base from March 9-12, 2020. Testing for SARS-CoV-2 was offered to passengers regardless of presence of symptoms. In total, 383 of 873 passengers agreed to provide nasopharyngeal swab (NP) specimens. A quality management system (QMS) was instituted to ensure samples were handled with an emphasis on safety and sample integrity.

As part of the QMS, all samples were triple-contained; NP specimens were placed in a sealed sample tube (primary container, PC) inside a biocontainment bag (secondary container, SC) before being wrapped with other SCs inside an absorbent waterproof under pad (AWU) inside a 95-kPa sealed bag (tertiary container, TC). SCs were placed in an AWU-lined sample cooler with ice packs for transport to the shipment sample processing center (SSPC). The SSPC was a converted hotel kitchenette consisting of a table for data entry, sample processing space on the floor, 4 minifridges to store samples at 4°C until shipment, and a connected living room serving as the staff resting and staging area. In the SSPC, the sample cooler was handed off to a processing officer who performed quality control (QC). QC consisted of (1) removing each sample bag from the cooler; (2) inspecting sample tubes for container integrity, proper labeling, and accuracy of identifying information; and (3) placing each sample with other checked samples in the TC. The only personal protective equipment (PPE) used when performing QC was nitrile gloves. In this case, the processing officer was a non-Centers for Disease Control and Prevention (CDC) responder with basic PPE training and no background in sample biocontainment and biosafety. A CDC Laboratory Subject Matter Expert (LSME) was on-site overseeing sample processing and PPE usage.

During QC, the processing officer removed a sample bag from the cooler, stood, and saw a drop of colored liquid fall into the cooler while noting that approximately 0.5 mL of viral transport media was inside the SC. The processing officer immediately placed the SC containing the spill back into the cooler, and without moving, notified the LSME. The LSME blocked entry into the area and had the processing officer remain in place while checking the officer’s clothing, shoes, and surrounding floor area for indication of spillage. Confirming no visible spillage, the processing officer removed their gloves and placed them into the contaminated cooler, washed their hands, and secured the area by physically blocking entry. The LSME contacted the CDC Infection Control Team Lead (ICTL) to discuss exposure risk and cleanup steps. By phone, the LSME and ICTL performed a risk assessment considering the location and volume of spill, staff present, and decontamination capability. The following cleanup plan was immediately implemented:

1. The LSME asked all nonessential personnel to leave the SSPC. Two team members volunteered to remain to assist the LSME.
2. The LSME and both assistants donned in order N95 respirators, face shields, gowns, and double gloved with nitrile gloves.
3. Cleanup roles:
  - The LSME decontaminated the potentially contaminated items and work areas. All decontamination was performed by wiping with Sani-cloth bleach disinfecting wipes^7^ (PDI, NJ).
  - The assistants stood directly on the other side of a half-wall separating the SSPC and staff staging area, handing items to the LSME and receiving waste for disposal.
4. The LSME removed the SCs from the larger TC and placed them back into the cooler, then decontaminated and discarded the used TC. The LSME then removed individual SCs for integrity inspection, decontamination, and placement on a clean AWU. When the suspect bag was removed, the LSME noted the PC appeared closed with the lid attached. However, the SC was not properly sealed, facilitating the external leak. The LSME placed the compromised tube and bag into a new sealable bag and discarded them per CDC guidelines.
5. In this same manner, the LSME removed, decontaminated, and placed all the contents of the cooler into a clean area for subsequent processing. No other samples leaked, but several had open SCs, which were sealed before placement in the clean area.
6. The LSME decontaminated the inside and outside of the cooler and moved it to the clean area.
7. The LSME decontaminated the working area.
8. The LSME and the 2 assistants doffed PPE in order gloves, gowns, face shields, and then N95 respirators and disposed of as per CDC guidelines.
9. Clean nitrile gloves were donned by 1 assistant, and the remaining samples were processed for shipment. All samples were kept on ice packs during cleanup and processing; temperature integrity was not considered compromised.

Risk mitigation strategies were initiated as a result of this incident, including: (1) when removing samples from the cooler, ensuring these be held closely over the opening of the cooler with QC inspection performed before moving the sample to the TC; (2) re-training sample collection staff to ensure the SCs are adequately sealed before transport; and (3) storing disinfecting wipes and additional PPE (gloves, N95 respirators, gowns, and face shields) in the sample processing area. Real-time biosafety and biocontainment training conducted by an LSME in the field before sample handling was deemed essential to remove any confusion and discomfort felt by staff when handling a potential infectious spill.

## Discussion

**Figure 1.**
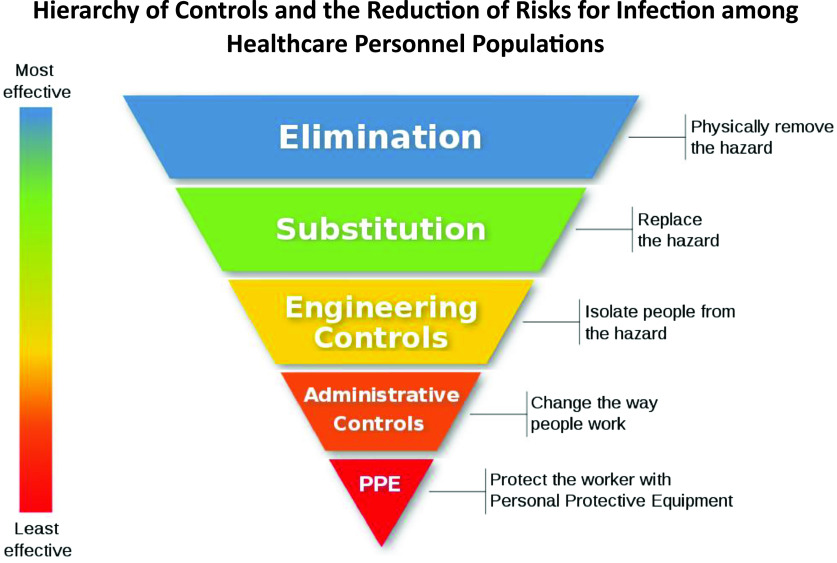
Hierarchy of controls and the reduction of risks for infection among healthcare personnel populations. Each level of the pyramid is associated with processes or functions that directly or indirectly protect staff from exposure or infection by infectious agents. When applied to sample acquisition for disease diagnostics (such as swabbing for SARS-CoV-2), while elimination or substitution stratagies are most effective at protecting health care workers, neither are possible in these scenarios so maximizing engineering controls, administrative controls, and PPE must be emphasized.

In the National Institute for Occupational Safety and Health (NIOSH) Hierarchy of Controls to reduce occupational hazards (Figure 1),^8,9^ the goal is to use the highest level possible of risk reduction. PPE is considered the least impactful level to reduce risk of infection, yet, before the incident, was the primary mitigation method used. The response plan for this incident effectively used 2 higher stages of risk reduction: Engineering Controls and Administrative Controls. Engineering Controls were implemented by removing all nonessential staff from the SSPC. Administrative Controls were implemented for sample processing by modifying where the SCs were handled (in this case, just over the opening to the sample cooler) and in conducting additional training with emphasis on ensuring SCs are properly closed. No cause for the tube leak was determined, but we hypothesize leaking occurred due to changes in pressure after changes in temperature. Further studies on this could help ensure safer packing and shipping of biohazardous samples. Proper closing of the PCs and visually inspecting them before placement in the SCs was emphasized.

There are 5 lessons from this incident that can be brought to the field on a larger scale. First, personnel in SSPCs should only include those directly processing and handling samples. This ensures there is no unnecessary exposure of people to potentially infectious agents. Second, emphasis on sealing the PCs and SCs at collection coupled with performing QC immediately over the sample cooler will minimize the likelihood, area, and severity of contamination from a spill. Third, as field-based sample collection increases outside of traditional clinical settings by staff who are not laboratorians, there is a need to have in place standardized, uncomplicated plans to train responders in biohazard mitigation and biocontainment practices. Fourth, field staff processing samples should use adequate PPE^10,11^ to reduce risk of exposure if an incident occurs. Lastly, to increase safety and ensure compliance with Category B shipping standards, it is important to ensure each field team has deployers appropriately trained in these processes.^12^ The incident at the quarantine facility demonstrated the necessity of keeping biohazard and biocontainment experts involved during all stages of sample handling and shipment. The LSME was able to provide accurate guidance on how to properly protect staff inside the processing center and to communicate both the immediate risks and mitigation steps. Ensuring adequate training in biohazard mitigation and sample biocontainment to those tasked with performing SARS-CoV-2 sampling and processing duties is essential to protect our frontline staff assisting in the COVID-19 response.
